# Antibacterial Effect of (2E,2E)-4,4-Trisulfanediylbis(but-2-enoic acid) against *Staphylococcus aureus*

**DOI:** 10.1371/journal.pone.0197348

**Published:** 2018-05-24

**Authors:** Tao Wu, Yina Huang, Yijun Chen, Min Zhang

**Affiliations:** 1 State Key Laboratory of Food Nutrition and Safety (Tianjin University of Science and Technology), Tianjin, China; 2 Key Laboratory of Food Nutrition and Safety, Ministry of Education, China; 3 Tianjin University of Science and Technology, Institute for New Rural Development, Tianjin, China; 4 Engineering Research Center of Food Biotechnology, Ministry of Education, Tianjin, China; Fujian Agriculture and Forestry University, CHINA

## Abstract

A new highly active molecule, (2E, 2E)-4,4-trisulfanediylbis(but-2-enoic acid) (TSDB), was designed and synthesized through comparative molecular field analysis with the diallyl trisulfide structure of garlic. TSDB exerted a strong inhibitory effect against *Staphylococcus aureus*, with minimal inhibitory and minimal bactericidal concentrations of 16 and 128 μg/mL, respectively. TSDB destructed the integrity of the *S*. *aureus* cell membrane but weakly damaged the bacterial cell wall. TSDB also increased the conductivity and protein expression in microbial broth but minimally influenced the level of extracellular alkaline phosphatase. TSDB could be a novel food preservative.

## Introduction

*Staphylococcus aureus*, an aerobic and facultative anaerobic Gram-positive bacteria, exists widely in nature and grows through various modes in food, mainly in dairy, egg, and cooked meat, and dairy-containing frozen products. Food-borne diseases caused by *S*. *aureus* causes massive economic losses to animal husbandry and food processing industry [1]. About 241,000 illnesses per year in the United States were caused by *S*. *aureus* [2]. Food-borne disease caused by S. aureus is still an important issue facing the food industry and consumers [3]. Therefore, new antibacterial products must be developed.

Many studies reported alliin exerts antibacterial action against *S*. *aureus*. Allicin has strong antibacterial effects on *S*. *aureus*, minimum inhibitory concentration is 32–64 μg/mL [4]. The main antimicrobial effect of allicin is due to its chemical reaction with thiol groups of various enzymes (e.g., RNA polymerase, alcohol dehydrogenase, and thioredoxin reductase), which can affect essential metabolism of cysteine proteinase activity [5–7]. However, allicin is unstable and easily converted into other sulfur compounds and antibacterial ingredients. Thus far, garlic and its derivatives have been compared in terms of microbial inhibition activity. Millet *et al*. compared the inhibition activity between allicin and its derivatives on spiral irritans [8]. Ren *et al*. [9] modified the structure of diallyl trisulfide (DATS) and synthesized five different compounds with superior antibacterial activities.

With the rapid development of molecular biology and computer technology, scholars have established three-dimensional quantitative structure–activity relationship (3D-QSAR), molecular docking, and homology modeling, for application in drug design, and pesticide, medicine, and food industries. 3D-QSAR methods can visualize areas that “favor” or “disfavor” the presence of a group exhibiting a particular physicochemical property. The comparative molecular field analysis (COMFA) method reveals the trends of quantitative changes in a material to avoid wastage in manpower and financial resources. Garlic and its derivatives have been compared in terms of microbial inhibition activity. However, the use of computer software models for comparison has not yet been reported.

**Fig 1:**
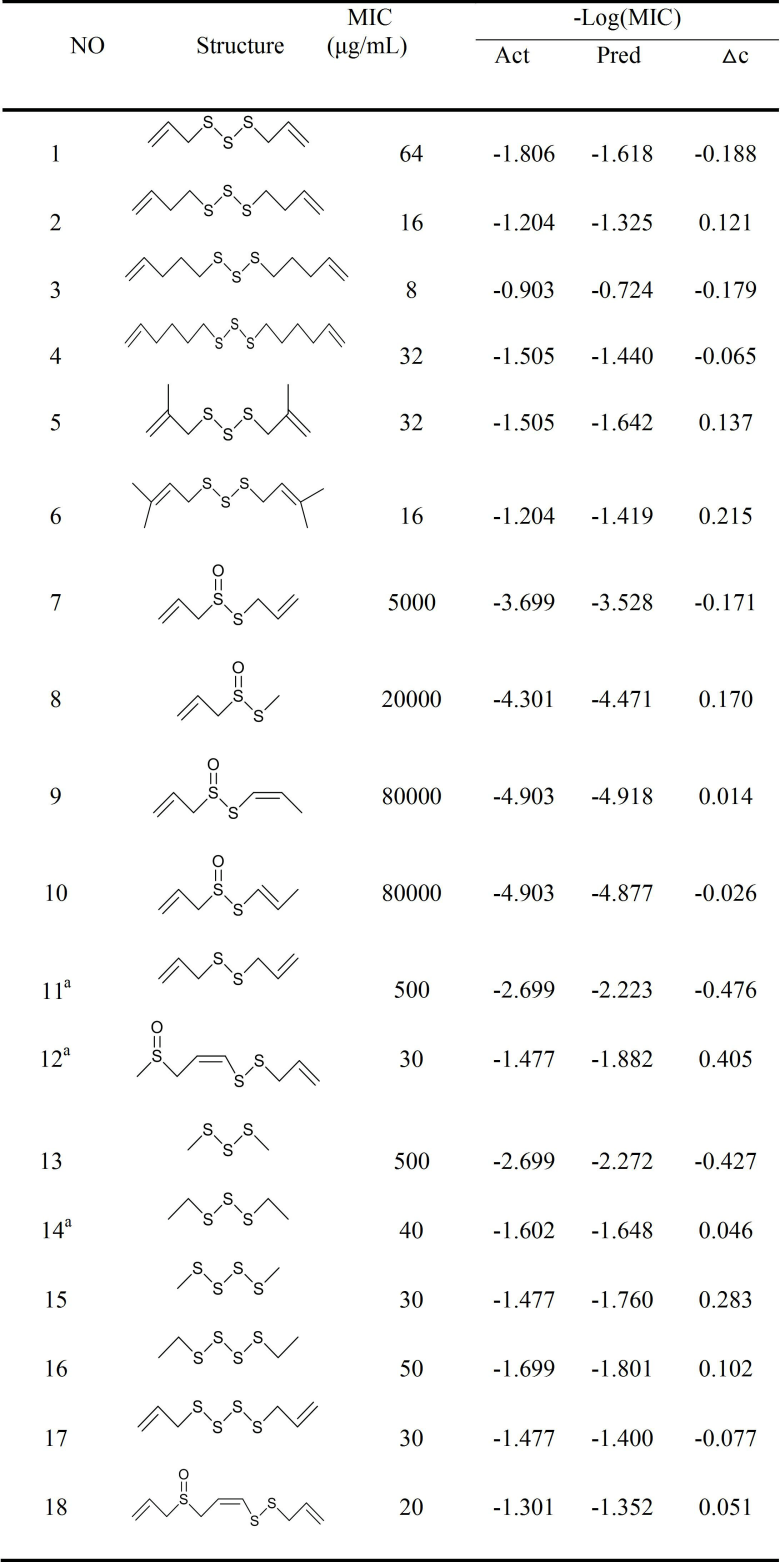
The structure and activity of selected compounds (-S-S- sulfides).

**Fig 2:**
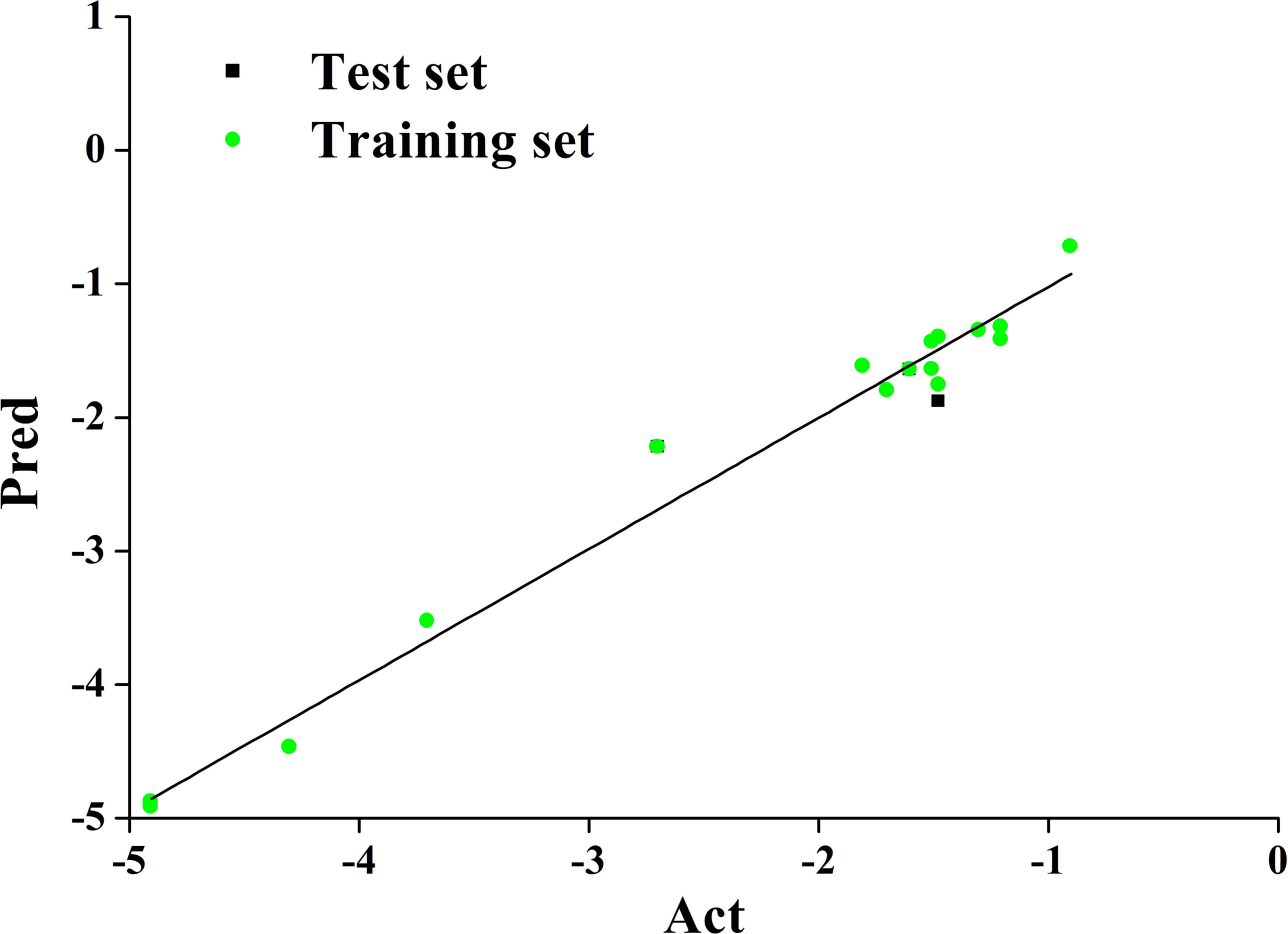
Diagram of training set and test set predicted activity versus actual activity.

We established a graphical model between biological activity and structure by determining 3D-QSAR among a series of garlic sulfide structures using the COMFA method. The model was then used to design compounds with high activity. The accuracy of the model was verified by experiments, and the underlying antibacterial mechanism was studied. The structure and activity of selected compounds (-S-S- sulfides) are illustrated in Fig 1 [9–12]. Sixteen compounds were randomly selected as a training set, and three other compounds were used as a test set. In the training set, compound 3 was used as template, and the other compounds were stacked with the corresponding atoms on the template compound skeleton by the alignment database module. The cross-validation coefficient (q^2^) and the regression coefficient (r^2^) of the QSAR model constructed by CoMFA are 0.618 and 0.983, respectively. The standard errors of estimate for the models is 0.203. (Fig 2) shows the correlation between the experimental and predicted values of activity in the training and test sets. The result indicates the high fit of the model.

**Fig 3:**
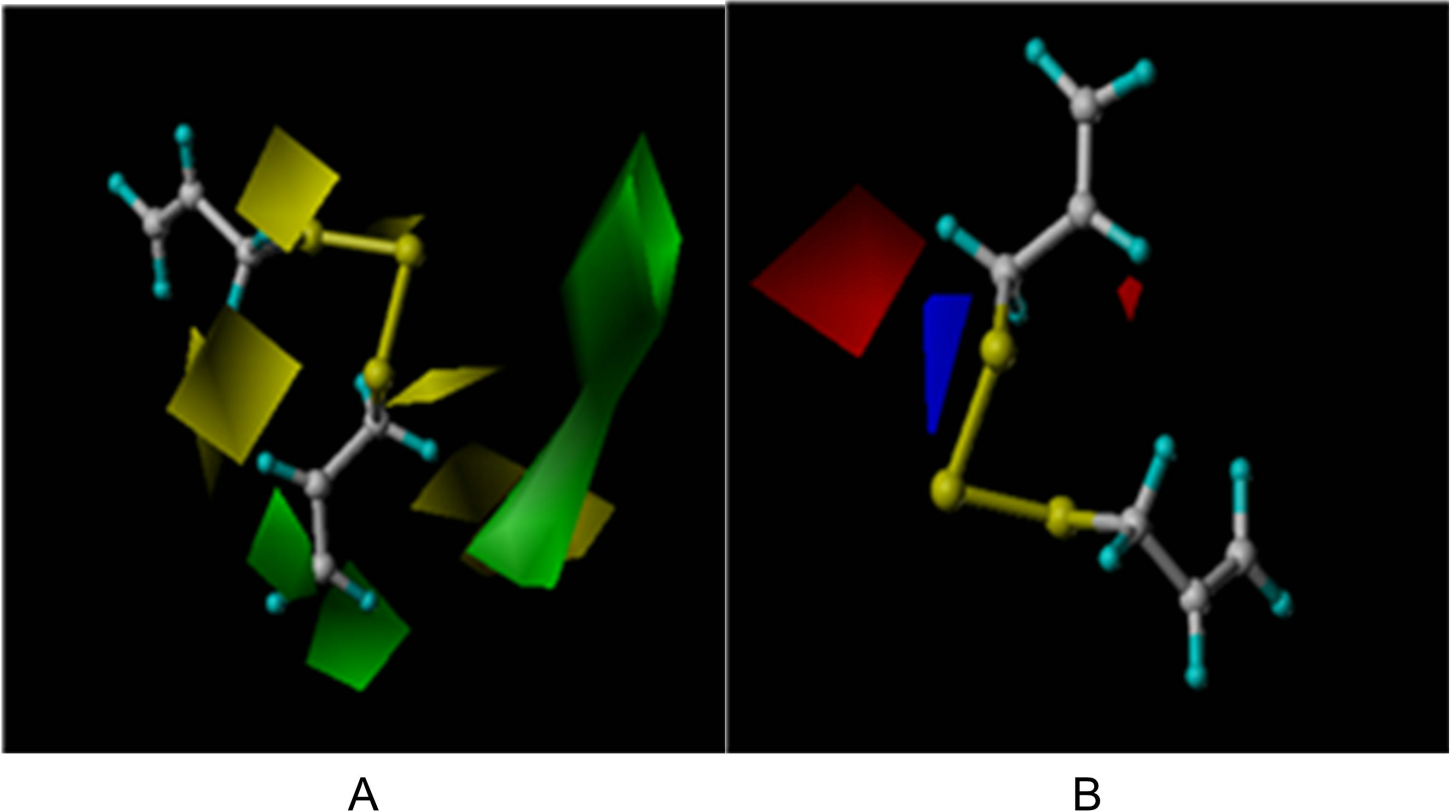
Equipotential diagram of COMFA model steric field and electroetatic. A, steric field; B, electroetatic field.

The COMFA coefficient map demonstrates regions of favorable and unfavorable steric and electrostatic contributions, providing structural insights into the activity of the studied compounds. Green represents more bulk being favored, yellow shows less bulk being favored, blue represents that positive potential is favored, and red indicates that negative potential is favored. According to analysis of the model structure (Fig 3), the double bond of compound 1 was modified to be economically efficient, with simplified synthesis.

## Methods and materials

### Materials

Models were analyzed with computer software: Chem Office; Discovery Studio2.5; SYBYL2.0. Both test compounds (HPLC purity≥95%) were purchased from Da Lian Yin Tai Technology company; *S*. *aureus* ATCC 26112 was obtained from the Culture Preservation Center, Tian Jin University of Science & Technology; alkaline phosphatase and protein quantitation kits were obtained from Jian Cheng Institute of Bioengineering (Nanjing, China). A Conductivity Meter (DDS-307) was used for electrical conductivity; optical density was measured by Microplate Reader.

### Computer simulation

We selected 19 compounds from the literature (Fig 2) [7, 9–11, 13]. The initial energy was first optimized using the MM force field in the Discovery Studio interface. Steepest-Descent and Powell methods were used to minimize optimization in the Sybyl X2.0 software. Simulated annealing algorithms were then used to search the global conformation and overlapping based on common framework. A COMFA model was calibrated according to the grid of 2.0 Å in all three dimensions within the defined region. The force field, including the steric and electrostatic field, used an sp3 carbon atom with +1.0 charge as a probe atom. The steric and electrostatic field energy was 90 kJ/mol, the filter column value was 2.0 kJ/mol, the regression analysis by partial least squares (PLS) established quantitative relationships, and then cross validation was used to obtain the best principal score. The non-cross-validated compounds were assessed by the variance r^2^, standard error of estimate (SEE), and F ratio.

### Minimal inhibitory (MIC) and minimal bactericidal concentration (MBC) determinations

The in vitro antimicrobial activity was assayed by the twofold broth dilution technique against Gram-positive bacteria (*S*. *aureus*) [14]. All test compounds were dissolved in ethanol and diluted into varying concentrations of 8 to 512 μg/mL; inocula was 1 × 10^5^ bacteria/mL. After incubation at 37°C for 24 h, the lowest concentration where no growth was observed was recorded as the MIC. For MBC testing, aliquots (0.1 mL) of broth from test tubes containing no growth were plated onto NB agar and again incubated overnight at 37°C. The highest dilution where survivors less than 5 CFU/mL were recorded as the MBC. Controls were performed using sterile NB without test compounds. All experiments were run in triplicate.

### Growth curves

The effect of test compounds on the growth of *S*. *aureus* was evaluated by a slightly modified method by Zeng [15]. *S*. *aureus* was cultured to logarithmic phase, experimental group contained MIC concentration of test compound, control group consisted of solution free of the test compound. The culture medium containing approximately 1 × 10^5^ cfu/mL *S*. *aureus* was further incubated (120 rpm at 37°C), and cell growth was monitored every 2 h at OD_600_nm, using a microplate reader.

### Electrical conductivity

The suspension of the tested bacteria was collected by centrifugation at 4000 rpm for 5 min, and the supernatant diluted by 20 times [11]. The diluent was monitored every 2 h using a conductivity meter to measure the electrical conductivity. The experiment was repeated three times.

### Determination of protein concentration

The suspension of the tested bacteria was collected by centrifugation at 4000 rpm for 5 min. Protein content of supernatant was determined by a slightly modified Coomassie Brilliant Blue method [16].

### Alkaline phosphatase assay

After the bacterial suspension was centrifuged at 4000 rpm for 5 min, the alkaline phosphatase (AKP) in the supernatant was determined as the change in absorbance at 405 nm over a 10-min period according to the kinetic method described by the International Federation of Clinical Chemistry [17].

### Statistical analysis

All experiments data were recorded as mean value ± standard deviation (SD). Statistical analysis was performed using SPSS19.0 software. Differences between means were considered statistically significant at p < 0.05.

## Results and discussion

**Fig 4:**
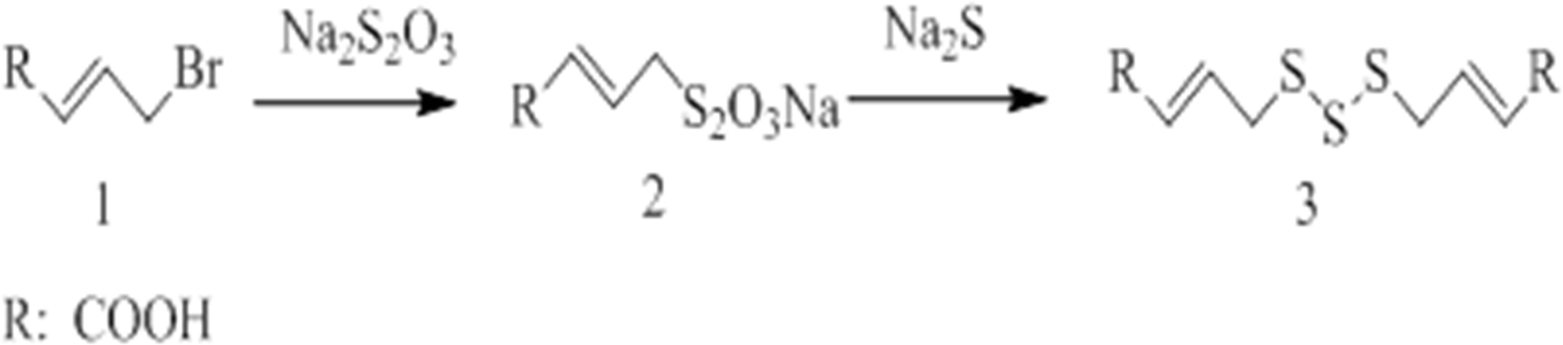
Routes for synthes of TSDB.

Our strategy for synthesis is demonstrated in Fig 4. Compounds were prepared by treatment of an unsaturated alkyl bromide with a saturated solution of sodium thiosulfate at 50°C—60°C. The product of salt 2 in the solution was added with sodium sulfide (1.0 equiv) to obtain 1,3-diallyltrisulfane derivative 3 at 95% yield after column chromatography. The product was characterized by 1H NMR spectroscopy(S1 Fig) [18].

DATS, an allicin breakdown product, is easily synthesized and more stable than the extremely volatile allicin. DATS is the principal antimicrobial compound in garlic [19]. (2E, 2E)-4,4-Trisulfanediylbis (but-2-enoic acid) (TSDB) belongs to the garlic family compounds. In this study, DATS and the derivative compound TSDB were assayed in vitro for antimicrobial activity against *S*. *aureus* ATCC26112. The MIC and MBC of the tested microorganisms were detected. Both of the compounds displayed good inhibition of the growth of Gram-positive bacteria *S*. *aureus* ATCC26112; the MIC values were 16 and 48 μg/mL, and MBC values were 128 and 256 μg/mL, respectively. TSDB exhibited higher activity than DATS because greater carboxyl bulk is favored, and the activity is enhanced. Carboxyl contributes positively to the activity because of the extremely bulky substituents and negative potential created by oxygen atoms. In summary, we demonstrated that TSDB with high antibacterial activity can become a new antibacterial agent.

Antimicrobial activity has been reported for several allyl sulfide compounds, involving the number of disulfide bounds in the order of DATTS > DATS > DADS > DAS [20]. Given the difficulty and high cost of synthesizing tetrasulfide, we selected trisulfide as the framework; the carbon chain length was not increased, but the antibacterial activity was markedly increased to 16 μg/mL. This finding showed that a COMFA model with good predictive power of antimicrobial activity against *S*. *aureus* was generated.

**Fig 5:**
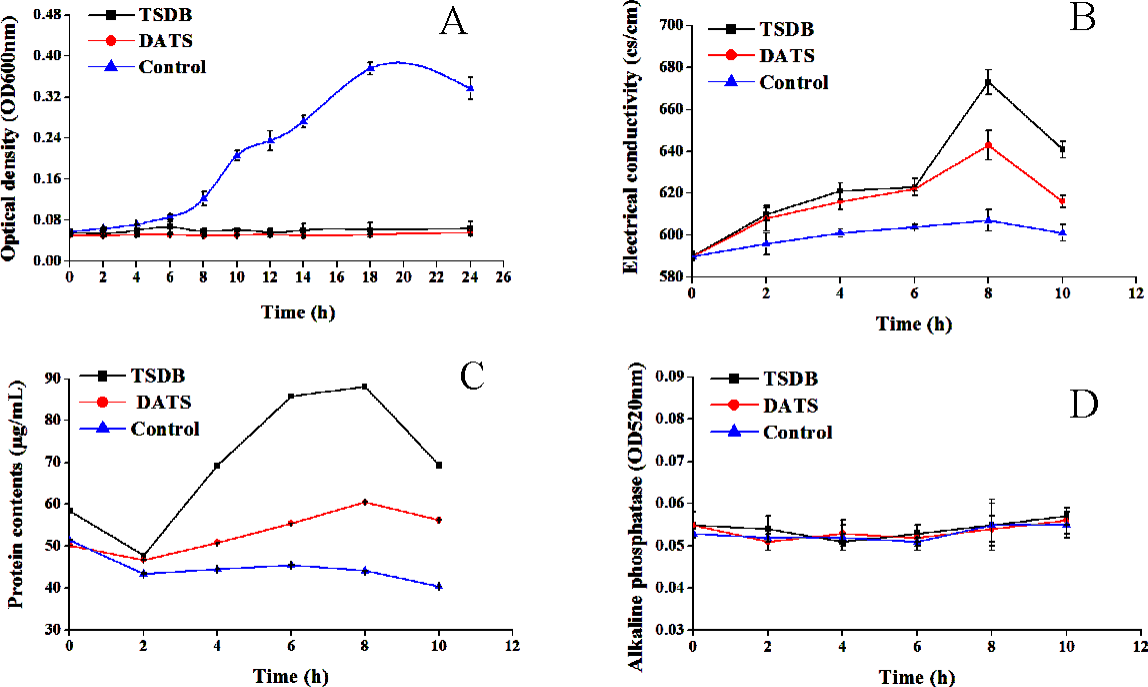
The antibacterial activity of both compounds against S. aureus. A, Effect of TSDB and DATS on cell growth; B, Effect of TSDB and DATS on Conductivity; C, Effect of TSDB and DATS on protein leakage; D, Effect of TSDB and DATS on Alkaline phosphatase.

The antibacterial activity of both compounds against *S*. *aureus* was further plotted by the time courses of *S*. *aureus* growth. As shown in Fig 5A., the growth of *S*. *aureus* was completely inhibited after 24 h of incubation at the MIC. Moreover, the growth curve results show that control cultures without TSDB and DATS undergo a lag phase in the first 2 h, gradually increase from 2 h to 18 h, and enter a decline phase after 18 h of cultivation. These findings further demonstrate that treatment with test compounds, even at low concentrations, can delay the growth cycle of *S*. *aureus* in a dose-dependent manner. Fig 5B shows increased electrical conductivity of the bacterial solution without the added TSDB and DATS, but the overall change is not significant. However, electrical conductivity slowly increased after TSDB and DATS action for 6 h, increased sharply to 673 and 643 cs/cm from 6 h to 8 h, being 4.88 times and 3.12 times the values of the control group, respectively. Conductivity treatment with TSDB was 1.57 times that of DATS, indicating that TSDB exhibits stronger cell membrane permeability than DATS. The tendency shows a certain decline after 8 h, possibly owing to damage to the cell membrane of *S*. *aureus* by TSDB and DATS, resulting in ion exudation. However, the residual surviving bacteria were in a state of stress, and ions can be finitely used to repair the cell membrane under certain conditions.

Fig 5C suggests that the protein contents in microbial broth decreased in the first 2 h after treatment with TSDB and DATS, increased to 88.19 and 60.50 μg/mL, respectively, and slightly decreased thereafter. At the beginning, the slow growth of bacteria will consume the protein in the culture. Then, with the pharmacological action of TSDB and DATS, the cell structure was destroyed, and the proteins flowed.

Membrane leakage may reduce the transmembrane proton electrochemical gradient and thereby deactivate energy-dependent reactions, such as ion transport, ATP synthesis, and metabolite sequestration [21]. This observation is consistent with the report that damage to the cellular wall and membrane can lead to the leakage of macromolecules and lysis [22]. We hypothesize that, through partial inhibition of DNA and protein synthesis, alteration of the electrochemical ability, the antibacterial activity of TSDB and DATS induce apoptosis in cells, affect microbial lipid biosynthesis, signal transduction, and react with thiol-containing proteins [23, 24].

Alkaline phosphatase is present between cell membrane and cell wall when the cell wall is destroyed and leaks out to the extracellular fluid with the increased permeability. Fig 5D illustrates that the extracellular alkaline phosphatase of the control group and the treatment group during the overall culture period was at a relatively low level. Considering that no difference was found between the treatment group and the control group, TSDB and DATS compounds demonstrated minimal influence on the tested strains of the cell wall.

Based on a COMFA model, TSDB, a compound with higher antibacterial activity, was designed and synthesized. TSDB displayed a strong inhibitory effect against *S*. *aureus*, with MIC and MBC values of 16 and 128 μg/mL, respectively. The accuracy and reliability of the COMFA model were illustrated, providing theoretical support for the research and development of new target drugs. The inhibitory effects of TSDB mainly occur through the destruction of cell membrane, increased permeability of the cell membrane, leakage of cellular contents, and altered normal metabolism inside and outside the cell, and ultimate inhibition of the growth of *S*. *aureus*. However, the antibacterial mechanism is complex, and further research involving gene levels and metabolomics are required.

## Supporting information

S1 Fig NMR spectrum of (2E, 2E)-4,4-trisulfanediylbis(but-2-enoic acid). (TIF)
