# Supplementary figures and images for: Antibacterial Effect of (2E,2E)-4,4-Trisulfanediylbis(but-2-enoic acid) against Staphylococcus aureus
Source: PLoS One. 2018 May 24;13(5):e0197348. doi: 10.1371/journal.pone.0197348 (PMC5967733; doi:10.1371/journal.pone.0197348)

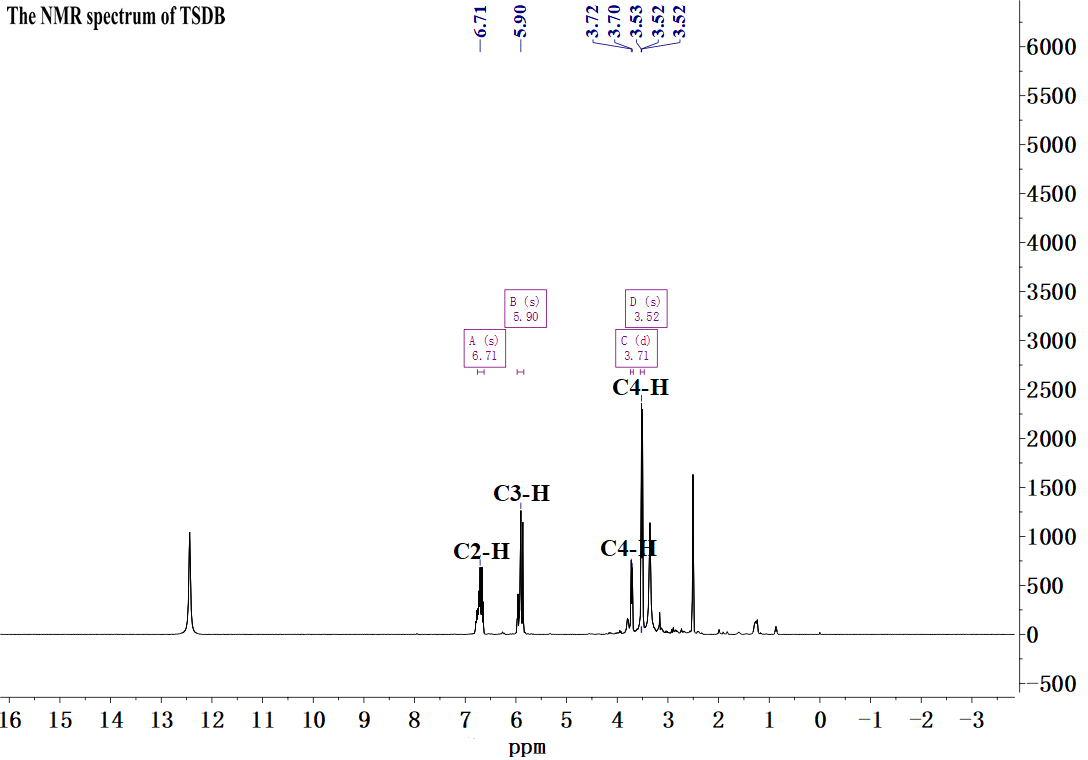

Supplement: S1 Fig — (TIF) [file pone.0197348.s001.tif]
